# Micro-Raman and micro-transmission imaging of epitaxial graphene grown on the Si and C faces of 6H-SiC

**DOI:** 10.1186/1556-276X-6-478

**Published:** 2011-07-29

**Authors:** Antoine Tiberj, Nicolas Camara, Philippe Godignon, Jean Camassel

**Affiliations:** 1 Laboratoire Charles Coulomb, UMR5221 CNRS-Université Montpellier II, Place Eugène Bataillon - cc074, 34095 Montpellier Cedex 5, France; 2 IMB-CNM-CSIC, Campus UAB Bellaterra, Barcelona 08193, Spain

## Abstract

Micro-Raman and micro-transmission imaging experiments have been done on epitaxial graphene grown on the C- and Si-faces of on-axis 6H-SiC substrates. On the C-face it is shown that the SiC sublimation process results in the growth of long and isolated graphene ribbons (up to 600 *μ*m) that are strain-relaxed and lightly p-type doped. In this case, combining the results of micro-Raman spectroscopy with micro-transmission measurements, we were able to ascertain that uniform monolayer ribbons were grown and found also Bernal stacked and misoriented bilayer ribbons. On the Si-face, the situation is completely different. A full graphene coverage of the SiC surface is achieved but anisotropic growth still occurs, because of the step-bunched SiC surface reconstruction. While in the middle of reconstructed terraces thin graphene stacks (up to 5 layers) are grown, thicker graphene stripes appear at step edges. In both the cases, the strong interaction between the graphene layers and the underlying SiC substrate induces a high compressive thermal strain and n-type doping.

## Introduction

Since the first report by C.V. Raman in 1928 [1,2], Raman spectroscopy has become increasingly popular in materials science and, especially, in semiconductor physics and microelectronics. Basically, Raman scattering probes the inelastic scattering of a monochromatic light (photons) by the lattice vibrations (phonons) in a solid. Since in a crystalline solid, the phonons are very sensitive to the internal and external perturbations, like doping and stress, the frequency of the scattered light (photons) is a local probe of the perturbation experienced (or not) by the medium. For a more detailed introduction and description of Raman and micro-Raman spectroscopy in materials science (and especially in semiconductors) please refer to [3-5].

Today numerous applications exist that cover the whole development of modern electronic and optoelectronic devices. They run from basic inspection of as-grown semiconductors to advanced device inspection tools. For instance, Raman mapping enables to check the crystalline quality [6,7], the composition [8,9], the doping level [10-13], or the uniformity of as-grown semiconductor materials. Along this line one on the most popular applications in microelectronics is strain measurements, either at the device or at the full wafer scale [9,14-17]. Raman measurements can also be used for final device inspection, through the temperature mapping of operating devices like FETs, lasers, and actuators [18-21]. In this case, thanks to the use of recent turnkey Raman systems, one can perform fast mapping with spatial resolution down to 300 nm. Combining with the use of several laser wavelengths, one can also probe the in-depth profile of multilayer systems and device. All together, these features confirm the unique versatiliy and potentialities of micro-Raman imaging in microelectronics. As a consequence, and because of its contactless and nondestructive nature, micro-Raman spectrosocopy (*μ*RS) has become an attractive characterization tool in industrial clean-room facilities.

In this field, graphene is a new comer. Because of its outstanding electronic, thermal, optical, and mechanical properties [22-24] it can be considered as a promising candidate for future carbon-based electronics [25]. However, and because of the so-called Kohn anomaly (which is nothing but the failure of the usual adiabatic Born-Oppenheimer approximation in zero-gap semiconductors [26]) it is also a perfect example to illustrate all the applications of *μ*RS that have been mentioned before. Raman spectroscopy on few layers graphene (FLG) not only can evaluate the crystalline quality but also, the thickness, the stacking order of graphene sheets, the doping level, and finally the residual strain.

In this study, we review some recent Raman imaging results collected on epitaxial graphene grown on the C- and Si-faces of 6H-SiC substrates [27-29]. In the first section, we briefly describe the growth techniques and the experimental set-up used for micro-Raman and micro-transmission imaging. In the second section, we discuss results collected on self-organized graphene ribbons grown on the C-face of 6H-SiC substrates and we show how thicknesses, stacking order, and a rough estimate of doping level can be obtained. Finally, in the third section, FLG grown on the Si-face are investigated and we show how the compressive stress experienced by such FLGs can be estimated.

## Experimental details

### Growth technology

All samples were grown using the processes described in [27-29]. We used 1 × 1 cm^2 ^pieces of on-axis 6H-SiC substrates cut from, either, the Si-terminated (0001) face or the C-terminated  face of 6H-SiC wafers. Before cutting, polishing was done by Novasic to get Epiready^® ^morphology [30]. Then, a sacrificial oxide was thermally grown and chemically etched in HF to remove any trace of sub-surface damage from the polishing process. Finally, standard RCA treatments were done to remove any trace of surface contamination. All treatments were clean-room compatible and similar to the one used for SiC before thermal oxidation or post-implantation annealing. In this way, atomically flat surfaces were systematically obtained.

For sublimation, we used a high temperature furnace from Jipelec [31] previously dedicated to post-implantation annealing. It was rf-induction heated and fitted with a turbo-molecular pump. The vacuum limit reached in this way was 10^-6 ^Torr. Before sublimation, the samples were heated at 1150°C for 10 min to remove any trace of native oxide. During the growth, the samples were covered by a graphite cap to increase the C and Si partial pressures over the SiC surface. Such graphite coverage lowers the Si out-diffusion process during the growth and enables to perform FLG growth at higher temperature. This promotes better SiC surface reconstruction.

In this way, on the C-face of 6H-SiC SiC substrates after 15 min annealing at 1700°C in a secondary vacuum, the growth of long (self-organized) graphene ribbons can be reached [27]. These ribbons are 5-*μ*m wide and 150-*μ*m long, but a longer 1700°C annealing results in longer ribbons (up to 600-*μ*m) with the same width. The width does not depend on the annealing time because the ribbons fully occupy a single terrace of the heavily reconstructed (step-bunched) SiC surface [28].

On the Si-face, to increase the FLG anisotropy, a modified growth process was used. The growth was done at 1750°C for 20 min under argon with a graphite cap covering the sample [29]. In this way, a full graphene coverage was obtained with a similar (pronounced) step-like morphology of the SiC substrate. The average terrace width was again 5 -*μ*m and the average step height 10 nm. Optical microscopy showed that the terraces had a remarkable homogeneity of width and orientation over a scale of 1 cm^2^.

### Coupling micro-Raman spectroscopy with micro-transmission measurements

Raman spectra were collected at room temperature, using a Jobin-Yvon Horiba T64000 spectrometer operated in the confocal mode. The 514-nm line of an Ar-Kr ion laser was used for excitation. With a × 100 microscope objective, the spot diameter was about approximately 1 *μ*m with, typically 1-mW power focussed on the sample. To combine micro-Raman spectroscopy with micro-transmission experiments, a low noise photodiode was inserted between the SiC substrate and the XYZ piezoelectric stage. For details, see [28]. In this way, it was possible to measure at the same time (using the same laser beam as probe) the power transmitted through the sample and the associated micro-Raman spectrum. The true FLG's spectra were obtained by subtracting the SiC reference signal from the experimental results.

The graphene extinction was deduced using the following equation:(1)

in which *T*_0 _is the bare SiC transmittance value and *T *the modified one with epitaxial FLGs on top. From the work of Ref. [32] it can be expressed as:(2)

In this equation, *n *= 2.68 is the SiC refractive index, *N *is the number of graphene layers, and *σ *is the optical conductivity of a single (isolated) graphene sheet that was confirmed experimentally [23]. The relative (theoretical) extinction of a monolayer and a bilayer graphene on top of a SiC substrate is then 1.23 and 2.44%, respectively.

## Raman imaging of isolated graphene ribbons grown on the C-face

**Figure 1:**
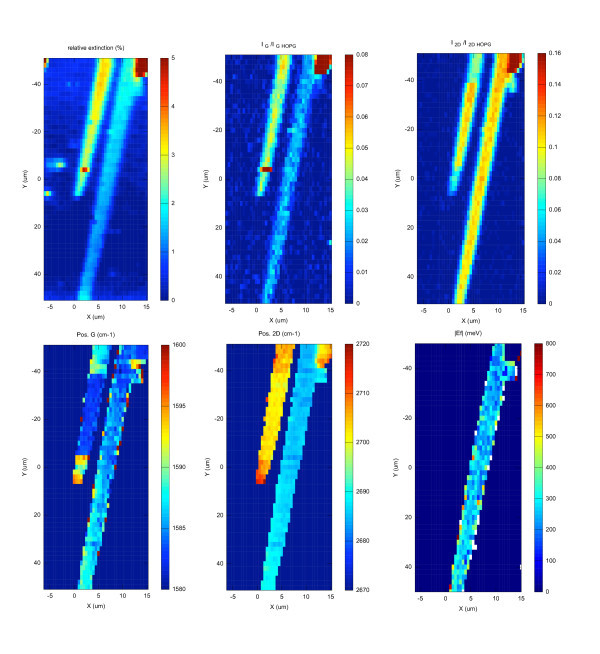
**20 × 100 *μ*m^2 ^maps of two graphene ribbons grown on the C-face of 6H-SiC**. The step sizes are 0.5 and 2 *μ*m for the *X*- and *Y*-axes, respectively. The relative extinction, the normalized intensities, and Raman shifts of the G and 2D band are shown. The right (left) ribbon corresponds to a monolayer (bilayer) graphene. The absolute value of the Fermi level is evaluated from the ratio between the intensities of the 2D and G bands only for the monolayer. It corresponds to a doping level between 3 × 10^12 ^and 9 × 10^12 ^cm^-2 ^with an average of 6 × 10^12 ^cm^-2^.

**Figure 2:**
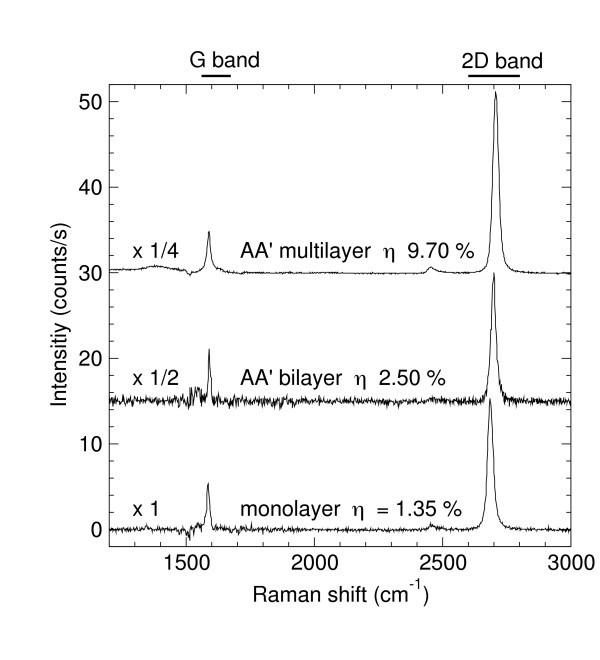
**Raman spectra of different ribbons: a monolayer, a misoriented bilayer, and a turbostratic multilayer with their corresponding relative extinction**. A misoriented bilayer has a similar Raman spectrum than a monolayer with a twice intensity. The multilayer corresponds to eight graphene sheets that are all disoriented with respect to each other. Therefore, the single Lorentzian shape of the 2D band cannot be used as a proof to assert the monolayer character of a FLG sample.

**Figure 3:**
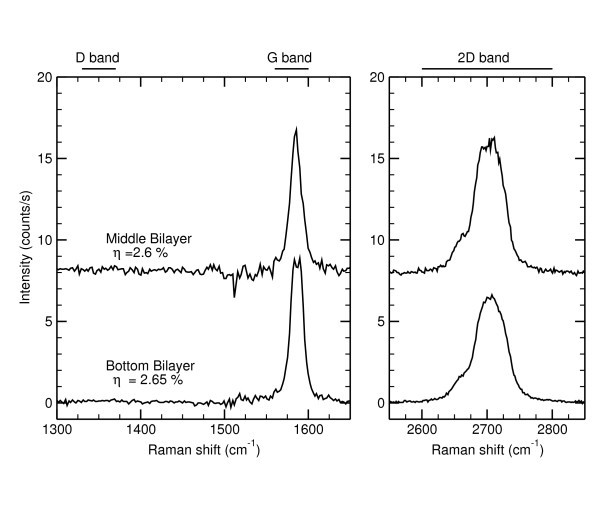
**Raman spectra of different area of the AB bilayer ribbon**. One is extracted from the bottom, the second from the middle of the ribbon. The bottom part of the ribbon exhibits a double G peak that can correspond to a different doping level between the top and bottom graphene sheet.

In Figure 1, we show the results of a large (20 × 100 *μ*m^2^) map collected on two neighboring graphene ribbons. The step size was 0.5 *μ*m for the *X *direction and 2 *μ*m for the *Y *direction. Six individual maps are shown. The first one corresponds to the extinction values, the second one to the integrated intensity of the G band normalized to the HOPG peak. The third one gives the normalized integrated intensity of the 2D band, while the fourth and fifth ones give the shift of the G and 2D bands, respectively. Finally, the last one corresponds to the absolute value of the Fermi level computed from the previous results. Of course, because of the limited range of the XY piezostage (100 × 100 *μ*m^2^) the two ribbons could not be completely probed. But a first point to be noticed is that, on both ribbons, no D band map could be given. This is shown in greater details in Figures 2 and 3 and demonstrates the excellent crystalline quality of these graphene samples.

Let us now consider everything more in detail. The first (extinction) map shows that both ribbons have an excellent thickness uniformity. However, and because the relative extinction is different, it shows also that the left one is thicker than the right one. Concerning the absolute values, from these extinction maps complemented by additional point by point measurements, we find that the thinner (right) ribbon corresponds with relative extinction values *η *ranging from 1.2 to 1.4%. This shows that we deal with a true epitaxial monolayer graphene (MLG) ribbon. To ascertain this result, a Raman spectrum collected at the center of ribbon is shown in Figure 2 (lower spectrum). It is clearly similar to the one reported in the literature [33] for monolayers graphene exfoliated on top of an oxidized silicon substrate and all spectra collected on the same ribbon exhibited the same Raman fingerprint.

Typically, the G band falls between 1583 and 1587 cm^-1^, with a FWHM of the order of 13 cm^-1^, while the 2D band ranges from 2682 to 2688 cm^-1 ^with a FWHM around 25 cm^-1^. This means that the graphene ribbons are strain free, unlike epitaxial graphene grown on the Si-face of SiC. This is confirmed by the presence of wrinkles evidenced by AFM [27]. For such graphene monolayers, the absolute value of the Fermi level (and the doping level) can be extracted from the ratio *I*_G_/*I*_2D _between the integrated intensities of the G and 2D band^a ^according to [34]:(3)(4)

In these equations, *γ_ep _*= 21 meV is proportional to the electron-phonon scattering rate [35], *v*_0 _= 10^6 ^ms^-1 ^is the electron velocity. The 0.06 factor is deduced from the function *f*(*r_S_*) [34] by using the effective dielectric constant *ε*_eff _= 5.33 for our graphene layer comprised between air and 6H SiC (*ε*_6H-SiC _= 9.66 [36]). The absolute value of the Fermi level was then evaluated for all data points collected on the right ribbon (see Figure 1), giving absolute values between 200 and 350 meV. It corresponds to a doping level between 3 × 10^12 ^and 9 × 10^12 ^cm^-2 ^with an average of 6 × 10^12 ^cm^-2^. We have not checked directly on the same ribbon but transport measurements performed on few similar ones [28] gave a hole concentration of 5 × 10^12 ^cm^-2^. According to the work of [37] such concentration is also in excellent agreement with the G and 2D band positions.

Concerning the second ribbon (on the left side), as already said we found a twice larger relative extinction. Ranging from 2.6 to 2.8%, this indicates a bilayer system. The relative extinction and the G band intensity both indicate that this ribbon is a bilayer with an excellent thickness uniformity. On the contrary, the 2D band intensity map in Figure 1 reveals sharp variations. Basically, the ribbon can be divided into three different domains, the top and bottom part having a less intense 2D band intensity than the middle one. Since these variations are correlated with shifts of the 2D and G bands positions, we assume that there are some doping level fluctuations. Indeed, it has been recently demonstrated that the G Raman band depends strongly on its electrostatic environment [38,39]. If the top graphene sheet has a different doping level than the bottom one, the doping difference changes the Raman shift and intensity of the G band. It also breaks the inversion symmetry and activates antisymmetric modes (that are usually Raman inactive). This results in a splitting of the G band which was observed experimentally [40-42]. Our interpretation is strengthened by the two Raman spectra shown in Figure 3. In both cases, the 2D band exhibits the characteristic shape of AB (Bernal) stacking [33] but the G band is different. On the upper spectrum a single G peak is observed while on the lower one (collected in the bottom part of the ribbon) a clear G band splitting shows that both layers are not evenly doped.

Bernal stacking is not that usual for epitaxial graphene grown on the C-face of SiC substrates. Most of the time the graphene planes are slightly misoriented, corresponding to turbostratic stacking. In this work, we also found misoriented ribbons (not shown). The presence of rotational stacking faults between the two (or more) successive graphene planes results in Raman spectra similar to the monolayer one as shown in Figure 2. The line shape is not modified. Simply the intensity increases as the number of graphene layers increases. In Figure 2, we mentioned the relative extinction measured on these ribbons. We found 2.5 and 9.7% that corresponds, respectively, to a bilayer and a 7 or 8 misoriented layers stack. These spectra correspond to FLG where all graphene sheets are disoriented with respect to each other. Therefore, the fact that the 2D band has a single Lorentzian shape can definitively not be used as a proof to assert the monolayer character of FLG flakes. The combination of *μ*RS with micro-transmission measurements appears then as a most necessary tool to discriminate (without any ambiguity) between true MLG and misoriented multilayers. Of course, to perform such reproducible intensity measurements, any laser power fluctuation has to be carefully corrected. In this work, this was done using an additional low noise photodiode that measured continuously the laser power during the Raman map acquisitions.

**Figure 4:**
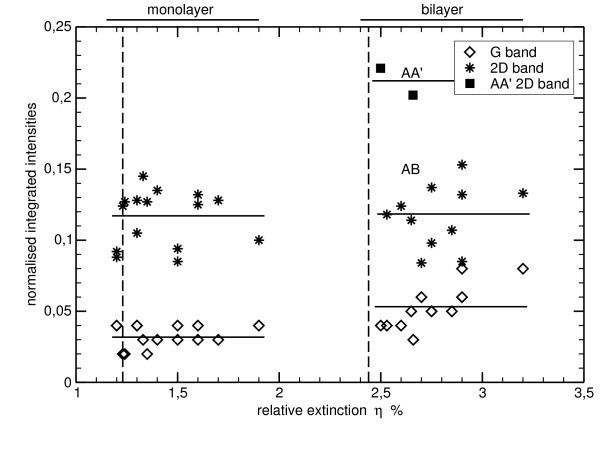
**Plot of the normalized integrated intensities of the G and 2D band against the relative extinction for several mono- and bilayer graphene**. The G and 2D band integrated intensities are normalized with the integrated intensities of an HOPG reference sample. Theoretical values of the relative extinction are indicated by two vertical dashed lines. A correlation exists between the G band intensity and the relative extinction. Two cloud of points can be easily distinguished between mono- and bilayers thanks to the relative extinction. The scattering of the G band intensity is too large to use it as an absolute thickness measurement. The 2D band scattering is even higher especially for bilayer. 2D band of AB bilayer is as intense as monolayers whereas misoriented bilayers are twice intense than a monolayer.

Coming back to the maps in Figure 1, some correlation exists between the extinction and the G band intensity. From this observation one could conclude that the G band intensity can be used to evaluate the number of graphene layers. This is not that simple. In Figure 4, we plot the normalized integrated intensities of the G and 2D band measured on many mono and bilayer graphene (not shown here) versus the corresponding extinction values. The theoretical extinctions values are indicated as vertical dashed lines. Despite the correlation existing between the G band intensity and the relative extinction, the scattering of the G band intensity is too large when compared to the difference between a mono- and bilayer. We can even find bilayers that has the same G band intensity than monolayers. Therefore, the G band intensity cannot be used as an absolute thickness measurement but rather as a first guess if the relative extinction cannot be measured. The relative extinction is indeed not measurable in several cases: (i) if no bare SiC exists on the sample (for instance as graphene fully covers the SiC surface), (ii) if impurities (dust) or metal contacts cover the graphene and/or the SiC. In this particular case, we can evaluate the thickness by assuming that the average G band normalized intensity of a monolayer is between 0.025 and 0.03. Beware that these values depend on the experimental configuration and must be calibrated. For thin FLG (¡5 layers) the error can be of 1 layer and, for thicker FLGs, the estimated thickness can have a factor two error. Unlike the G band, the 2D band intensity cannot be used to evaluate the thickness for several reasons. For monolayers, the 2D band intensity strongly depends on the Fermi Level [34]. We used this dependance to evaluate the absolute value of the doping in the monolayer ribbon. Moreover in Figure 4, we can clearly see that the 2D band of an AB bilayer is as intense as the one of a monolayer.

## Raman imaging of FLG grown on the Si face

**Figure 5:**
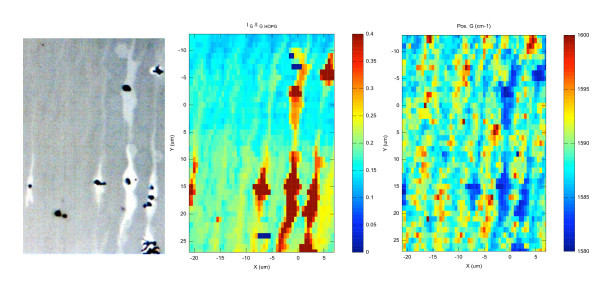
**30 × 40 *μ*m^2 ^optical image of the graphene surface and the corresponding Raman maps of the G band intensity and Raman shift**. The intensity of the G band is integrated and normalized by the G band of an HOPG reference sample. A full graphene coverage of the surface is observed with thickness inhomogeneities. FLG are thicker at the step edges (about 11 layers) than in the middle of the terraces (about 5 layers). On the edges we can clearly observed stripes: bright areas on the OM image and red areas on the G band intensity map. On the OM image, we can also see black points that correspond to C-rich graphite pits induced by an increased growth rate due to the presence of crystalline defects. On the G band intensity map, blue points mark the presence of Si clusters where the Raman fingerprint of silicon was observed. Finally, the G band is shifted to higher frequencies indicating that FLG are compressively stressed. This stress is progressively relaxed as FLG are thicker.

In Figure 5, we show a 30 × 40 *μ*m^2 ^micro-Raman map collected on an epitaxial graphene stack grown on the Si-face of a 6H-SiC substrate. The normalized integrated intensity of the G band is compared to its Raman shift and to an optical microscopy image (OM) of the same area. On the OM image, dark areas correspond to central part of the terraces, while the bright areas correspond to the edges of the step bunched SiC reconstructed surface. As already said, the terraces are 5 *μ*m wide and 10 nm high and, from the G band intensity, we find that graphene covers all the SiC surface. It is then impossible to measure directly the relative extinction and (consequently) the FLG thickness. Of course, a thickness estimate can still be done from the G band intensity. We found about 5 layers in the center of terraces (green-blue areas in Figure 5) and about 11 layers on the stripes close to the edge of terraces. On these stripes, we could also distinguish some black points on the OM image. At these points, the G band intensity is much intense which suggests that they correspond to thick graphitic pits. The Raman spectra of these pits exhibit a strong D band, characteristic of a bad crystalline quality. Such pits are probably induced by an increased growth rate coming from the presence of structural defects, like threading dislocations, in the SiC wafer.

**Figure 6:**
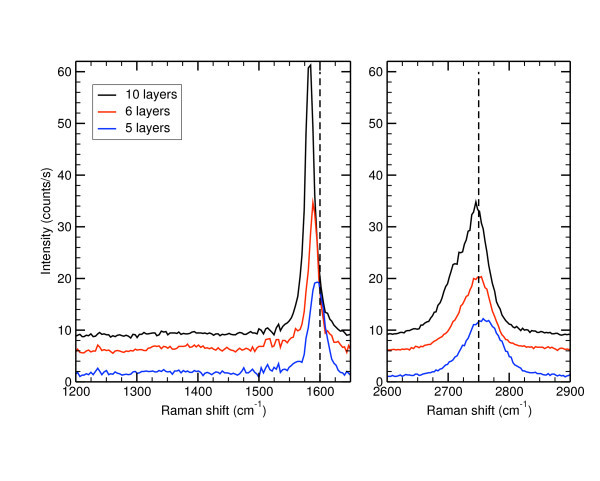
**Raman spectra collected in the middle of the terraces (5 to 6 layers) and on the stripes close to the step edges (11 layers)**. No D band can be observed confirming the excellent crystalline quality of these FLG. The asymmetric shape of the 2D band (that is more pronounced for the thicker FLG) reveals a Bernal stacking of the graphene planes. Finally, both bands are blue-shifted. Such shift can only be explained by a compressive strain of the graphene lattice coming from the differential dilatation during the cooling down of the sample. A partial strain relaxation occurs for thicker FLG since the thicker the less shifted.

In Figure 6, we gathered Raman spectra collected in the middle of the terraces and on the stripes. Unlike the graphitic pits, no D band can be observed. This shows that most of the grown FLG have an excellent crystalline quality. The 2D band are broad with a lower frequency shoulder. This shoulder is more pronounced for the thickest FLG which have a 2D band shape similar to the HOPG one. This asymmetric 2D band is a clear indication of Bernal stacking even for the thinner FLG where the low-frequency shoulder is known to become less visible [43].

These Raman spectra also reveal that both G and 2D bands are shifted to high frequencies. For the thinnest FLG the G band falls between 1590 and 1600 cm^-1 ^and the 2D band between 2750 and 2760 cm^-1^. Such high blueshift cannot be explained by doping but rather by a compressive stress experienced by the FLGs. This stress comes from the differential dilatation coefficient between the FLG and the SiC substrate when cooling down the sample after the growth. Thanks to the Grüneisen parameters that have been recently measured [24,44], this biaxial stress/strain can be estimated from the coefficients listed in Table 1. We find strain values between -0.3 and -0.4%, corresponding to local stress values ranging from -3 and -5 GPa. However, for thicker FLGs, the G and 2D band are less shifted. This reveals a partial strain relaxation as the graphene thickness increases, which can be easily seen in Figures 5 and 6. The thicker the FLG, the less blue shifted the spectrum.

**Table 1:** Frequency shifts of the G and 2D bands for a biaxial strain of 1% or a biaxial stress of 1 GPa [44,24].

*ε*_biax _= 1%	*σ*_biax _= 1 GPa
	
	

From these results, we confirm that there is a strong difference between graphene grown on the C-face and graphene grown on the Si-face of SiC substrates. On the Si-face graphene strongly interacts with the underlying SiC lattice. This interaction leads to the formation of the so-called buffer layer, which is covalently bound to the SiC lattice [45,46]. This buffer layer interacts also with the graphene layers that are grown subsequently. It induces a downward shift (*E*_D _= -0.4 eV) of the K point corresponding to a n-type doping. This interaction causes also deviations from the linear band dispersion leading to a parabolic dispersion with an apparent gap of approximately 0.25 eV. This strong interaction is also responsible for the thermal stress experienced by these graphene layers. This is no longer true on the C-face, on which it has been shown that graphene interacts very weakly with the underlying substrate [47,48]. It is the weakness of this interaction that explains why rotational stacking faults can easily occur and why the graphene sheets can relax the thermal stress by forming wrinkles or pleats.

**Figure 7:**
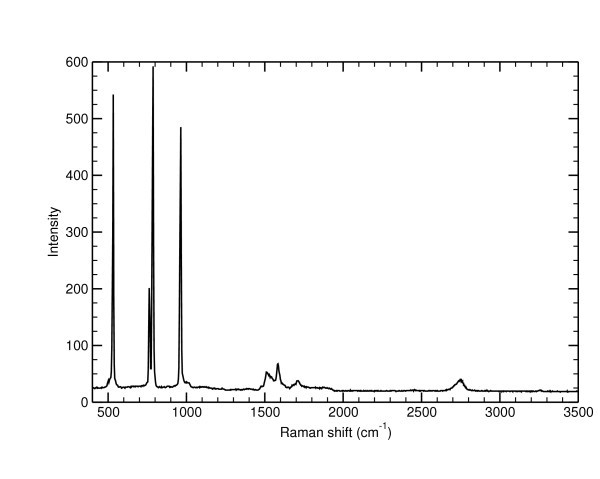
**Uncorrected Raman spectrum extracted from the Raman mapping that corresponds to one of the blue point in the G band intensity map**. The first-order Raman scattering of SiC correspond to the bands at 764, 786, and 964 cm^-1^. Its second overtone falls between 1400 and 2000 cm^-1 ^with the sharp G band around 1590 cm^-1^. The 2D band is around 2780 cm^-1^. No D band can be seen on this point. The sharp and intense band around 532 cm^-1 ^correspond to a crystalline Si cluster that is highly compressively stressed by the SiC substrate.

Finally, on the G band intensity map we can see several points marked in blue. These blue points correspond to area where crystalline silicon clusters were found. One of the corresponding uncorrected Raman spectrum is shown in Figure 7. The presence of these crystalline silicon (c-Si) clusters is evidenced by the sharp and intense band around 532 cm^-1 ^blue shifted compared to bulk silicon. This high blue shift is again due to a strong compressive stress induced by the SiC substrate (-2 GPa or a strain of -1.3%). First-order Raman scattering of the SiC substrate^-1 ^and the corresponds to the two TO modes of E_2 _symmetry at 764 and 786 cm^-1 ^and the A_1_(LO) phonon at 964 cm^-1^. Its second overtone with its characteristic fingerprint [49] falls between 1400 and 2000 cm^-1 ^under the sharp G band of FLG around 1590 cm^-1^. The 2D band is around 2780 cm^-1^. No D band can be seen on these points. Moreover, we can see that no significant variations of the G band intensity and Raman shift can be observed close to these Si clusters. This means that these Si clusters do not alter the graphene growth. These clusters are located close to the step edges, like the graphitic pits. There might be a link between the presence of different defects at the step edges like these clusters, the higher growth rate and the clear electrical anisotropy that has been evidenced by magnetoresistance experiments performed on several Hall bars with different orientations [29].

## Conclusions

Reviewing recent Raman imaging experiments performed on epitaxial graphene grown on the C and Si face of 6H SiC substrates, we have shown the benefits of combining Raman spectroscopy with micro-transmissions measurements. Provided the relative extinction of FLGs can be obtained, this enables to determine (without any ambiguity) the thickness, homogeneity, and stacking order (Bernal or turbostratic) of FLGs. On the C-face of SiC substrates we have shown that long, self-ordered, graphene ribbons can be grown. These ribbons have excellent crystalline quality and are strain relaxed. They are up to 600-*μ*m long and 5-*μ*m wide. They are mainly monolayers and Bernal stacked bilayers but turbostratic bi and multilayer areas have also been found. Finally for monolayers graphene, we also illustrated how the absolute value of the Fermi level can be found, in good agreement with electrical results.

On the Si face, on the opposite, a full graphene coverage of the SiC surface has been found. The surface is still heavily step-bunched but a high compressive thermal strain and n-type doping has been observed. It confirms that on the Si face a strong interaction exists between the graphene layers and the underlying SiC substrate. FLG on the Si-face exhibits Bernal stacking with thickness inhomogeneity. Thin (5 layers) FLGs were grown in the middle of terraces, while thicker graphene stripes grew close to the step edges. In the vicinity of these steps disordered graphite pits and crystalline Si clusters were found. There might be a link between the presence of these defects, the thickness inhomogeneity and the clear electrical anisotropy that has been recently evidenced by magnetoresistance experiments.

## Competing interests

The authors declare that they have no competing interests.

## Authors' contributions

NC and PG prepared the samples. AT carried out the micro Raman spectroscopy and microtransmission experiments. AT and JC performed the analysis of the Raman and transmission data. AT wrote the manuscript. All authors read and approved the final manuscript.

## Endnote

^a^The ratio between the integrated intensities of the 2D and G band depends on the experimental setup. In our case *I*_2D _= *I*_G _= 1.02 for our HOPG reference sample. For the lack of a better knowledge, we assume that our setup is similar to the ones of Refs. [34,35].
